# No Country for Old Worms: A Systematic Review of the Application of *C. elegans* to Investigate a Bacterial Source of Environmental Neurotoxicity in Parkinson’s Disease

**DOI:** 10.3390/metabo8040070

**Published:** 2018-10-29

**Authors:** Kim A. Caldwell, Jennifer L. Thies, Guy A. Caldwell

**Affiliations:** 1 Department of Biological Sciences, The University of Alabama, Box 870344, Tuscaloosa, AL 35487, USA; Jthies@crimson.ua.edu (J.L.T.); gcaldwel@ua.edu (G.A.C.); 2 Departments of Neurology and Neurobiology, Center for Neurodegeneration and Experimental Therapeutics, Nathan Shock Center for Research on the Basic Biology of Aging, University of Alabama at Birmingham School of Medicine, Birmingham, AL 35294, USA

**Keywords:** neurodegeneration, Parkinson’s disease, *C. elegans*, *Streptomyces venezuelae*, natural product

## Abstract

While progress has been made in discerning genetic associations with Parkinson’s disease (PD), identifying elusive environmental contributors necessitates the application of unconventional hypotheses and experimental strategies. Here, we provide an overview of studies that we conducted on a neurotoxic metabolite produced by a species of common soil bacteria, *Streptomyces venezuelae (S. ven*), indicating that the toxicity displayed by this bacterium causes stress in diverse cellular mechanisms, such as the ubiquitin proteasome system and mitochondrial homeostasis. This dysfunction eventually leads to age and dose-dependent neurodegeneration in the nematode *Caenorhabditis elegans*. Notably, dopaminergic neurons have heightened susceptibility, but all of the neuronal classes eventually degenerate following exposure. Toxicity further extends to human SH-SY5Y cells, which also degenerate following exposure. Additionally, the neurons of nematodes expressing heterologous aggregation-prone proteins display enhanced metabolite vulnerability. These mechanistic analyses collectively reveal a unique metabolomic fingerprint for this bacterially-derived neurotoxin. In considering that epidemiological distinctions in locales influence the incidence of PD, we surveyed soils from diverse regions of Alabama, and found that exposure to ~30% of isolated *Streptomyces* species caused worm dopaminergic neurons to die. In addition to aging, one of the few established contributors to PD appears to be a rural lifestyle, where exposure to soil on a regular basis might increase the risk of interaction with bacteria producing such toxins. Taken together, these data suggest that a novel toxicant within the *Streptomyces* genus might represent an environmental contributor to the progressive neurodegeneration that is associated with PD.

## 1. Genetics of Parkinson’s Disease

As the second most common neurodegenerative disorder, Parkinson’s Disease (PD) is considered a disease of aging, since it primarily affects individuals over the age of 65. It is characterized by a progressive loss of dopaminergic (DA) neurons in the substantia nigra pars compacta and results in resting tremors, muscle rigidity, and impaired balance. Current treatments provide limited, and purely symptomatic, relief.

Several cellular processes have been associated with PD, including DA chemistry imbalances, abnormal vesicular trafficking, proteasome dysfunction, disrupted protein homeostasis, mitochondrial impairment, and impaired autophagy. While these are often examined as separate processes, it is likely that once a single inciting molecular event occurs, the pathogenic process encompasses overlapping molecular mechanisms. Advances have been made in understanding the processes that are associated with neurodegeneration through the analysis of gene mutations that are more commonly associated with autosomal-dominant [α-synuclein (PARK1/4) and LRRK2 (PARK8)] and recessive forms of familial PD [parkin (PARK2), PINK1 (PARK6), DJ-1 (PARK7), and ATP13A2 (PARK9)]. The gene products encoding many of these familial mutations have been implicated in cellular pathways involved in PD pathological disease mechanisms. However, even the most common gene mutation, LRRK2 (G2019S), is associated with <2% of identified PD cases [1]. In total, genetics account for ~10% of diagnosed PD, and it has become apparent that investigation into purely genetic factors will not elucidate all or even most PD incidence.

## 2. Environmental Factors Impacting Neurodegeneration

Recent research suggests that since familial forms of PD are fairly rare, environmental determinants may significantly contribute to the onset of neurodegenerative pathology. In addition to aging, one of the few established and reproducible epidemiological contributors to PD appears to be a rural lifestyle, where drinking well water, farming, and exposure to pesticides or herbicides may all be risk factors [2,3,4,5,6,7,8]. The herbicides (paraquat) and pesticides (rotenone) that are used in farming result in the formation of excessive reactive oxygen species (ROS). These both induce Parkinsonian phenotypes in animals [9], and rotenone also inhibits mitochondrial complex I [10]. Mitochondrial defects also are a common theme in PD pathogenesis. Mutations in the autosomal recessive familial genes, PINK1 and parkin, result in mitochondrial dysfunction [11]. However, it is important to note that the levels of herbicide and pesticide exposures that are often encountered in farming cannot completely account for the increased PD odds ratio for those living in rural areas [12]. With this information, we sought to identify an alternative environmental exposure that could partially account for the enhanced PD risk associated with rural living.

## 3. Soil Bacteria and Neurodegeneration

The gap in our understanding between environmental and inherited causes of PD remains long unresolved. Our ability to successfully reduce PD among susceptible individuals is dependent upon knowledge about factors that render certain populations at risk. The higher incidence of PD in rural areas, where the disease may actually be underreported due to health care disparities, remains a rare clue to a potential environmental contribution to the disease. Lifestyle and occupational distinctions among individuals from rural areas may present a more consistent exposure to terrestrial environmental factors that are simply less common in more developed lands and cities. For example, living on dirt floors, drinking well water, farming, and general interaction with soil microbes may represent a source of risk to certain individuals. Approximately one million distinct microbial species are estimated to comprise the approximately one billion microorganisms in a single gram of soil [13,14]. *Streptomyces*, a bacterial genus within the order Actinomycetales, is ubiquitously prevalent in soil samples (~6% of the microbial population) [15]. Notably, *Streptomyces* are renowned as a source of secondary metabolites; the genus includes over 70% of known antibiotics [16]. Therefore, we surmised that a putative source of the undefined environmental contributors to PD onset and progression could come from exposure to these common soil bacteria. We further hypothesized that exposure to a potentially neurotoxic compound of bacterial origin could be exacerbated by factors influencing genetic pre-susceptibility to neurodegeneration (or PD).

Proteasome inhibitors, many of which are isolated from Actinomycetes, have been shown to induce neurodegeneration in animal model systems. At least four characterized proteasome inhibitors are products of *Streptomycetes* isolated from soil, including lactacystin [17]. The selective loss of DA neurons after the systemic administration of epoxomicin, which is a naturally occurring proteasome inhibitor, or PSI ((Z-lle-Glu(OtBu)-Ala-Leu-al), which is a synthetic proteasome inhibitor, to rodents was reported [18]. While promising, these data proved to be difficult to reproduce by other research groups [19,20,21]. Nevertheless, the reliable progressive degeneration of dopaminergic neurons was, in fact, achieved via the direct administration of either of these agents into rodent brains [22]. Another intriguing study demonstrated that mice injected with a pathogenic actinomycete that is found commonly in soil and water*, Nocardia asteroides*, developed symptoms that phenocopied PD; moreover, these infected animals responded positively to the administration of levodopa [23]. Although the relevant mechanism of action remains unknown, subsequent in vitro and in vivo studies showed that infection with GUH-2, a specific strain of *N. asteroides*, resulted in the apoptotic death of substantia nigra DA neurons [24]. Further studies with GUH-2 revealed that it was neuroinvasive in both mice and monkeys, and that their brain infections resulted in the apoptotic death of DA neurons due to proteasome inhibition [24,25,26,27,28]. While these data have heightened speculation that bacterial exposure/infection could be a potential risk factor for PD, progress in this area of research has been limited. Taken together, we decided to ask if common soil bacteria from the genus *Streptomyces* could produce neurodegenerative secondary metabolites.

## 4. Using *C. elegans* to Model Neurodegenerative Phenotypes

**Figure 1:**
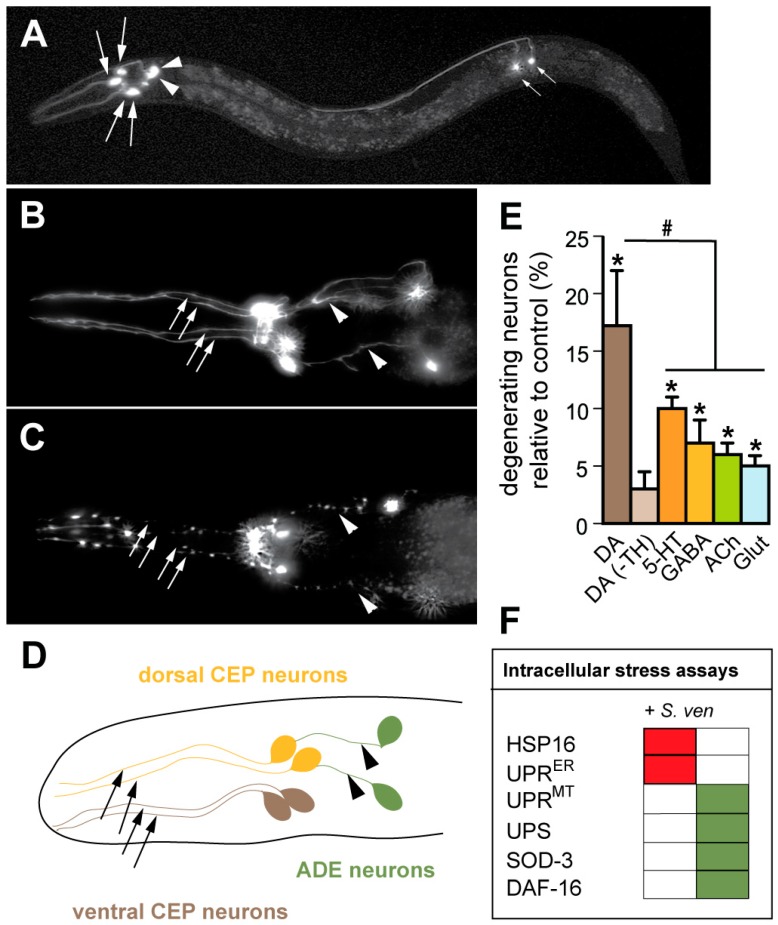
Exposure to the *S. venezuelae* metabolite causes dopaminergic neurodegeneration and intracellular stress in *C. elegans*. (**A**) Dopaminergic cell bodies and processes are illuminated using green fluorescent protein (GFP) driven from the dopaminergic (DA) transporter promoter (P*_dat-_*_1_::GFP). The six anterior DA neurons that are shown on the left side of this image include two pairs of cephalic neurons (CEPs, large arrows indicating cell bodies) and one pair of anterior deirid neurons (ADEs, arrowheads indicating cell bodies). There is also one pair of posterior deirid neurons (PDEs, small arrows indicating cell bodies); (**B**) Close-up of the anterior region of *C. elegans* where the six most anterior DA neurons are highlighted by GFP (P*_dat-_*_1_::GFP) with the four CEP neurons (arrows indicating neuronal processes) and two ADE neurons (arrowheads indicating neuronal processes) highlighted; (**C**) A worm expressing GFP in DA neurons displays neurodegenerative changes in all six anterior DA neurons following nine days of exposure to the *S. venezualae* (*S. ven*) metabolite; (**D**) Drawing of the *C. elegans* anterior DA neurons. Precisely six DA neurons in the anterior of *C. elegans* are found in pairs defined as two dorsal CEPs, two ventral CEPs, and two ADE neurons. The dorsal CEPs are post-synaptic to the ADEs, and are connected within this circuit, whereas the ventral CEPs and the ADEs do not display connectivity; (**E**) Separate neuronal subtypes within isogenic populations of worms were scored for neurodegeneration in animals where GFP was exclusively expressed to illuminate either the dopaminergic (DA) [+ and − tyrosine hydroxylase (TH) expression], serotonergic (5-HT), GABAergic (GABA), cholinergic (ACh), and glutamatergic (Glut) neuronal subclasses. All of the neuronal classes that were examined exhibited significant neurodegeneration following eight days of exposure to an *S. ven* conditioned medium, except animals wherein the DA neurons were devoid of TH through a genetic mutation (*cat-2*) (* *p* < 0.01; ANOVA). Significantly, the DA neurons displayed increased degeneration compared to all other neuronal classes (# *p* < 0.05; one-way ANOVA). The amount of neurodegeneration that was observed in animals exposed to an *E. coli* control conditioned medium was used as a baseline for standardization. To compensate for distinct neuronal classes containing different numbers of neurons, the percentage of total degenerating neurons (not worms with degeneration) was used for comparisons; (**F**) Intracellular stress response summary following exposure to *S. ven* metabolite. Gene reporters for stress assays tested are shown here. HSP16 is a homolog of the hsp16/hsp20/alphaB-crystallin family of heat shock proteins. The endoplasmic reticulum (ER) unfolded protein response (UPR^ER^) was assessed by measuring P*_hsp-_*_4_::GFP in *C. elegans*; HSP-4 is homologous to the mammalian ER chaperone, BiP. The mitochondrial unfolded protein response (UPR^mt^) was measured via P*_hsp-_*_6_::GFP; HSP-6 in *C. elegans* is a transcriptional reporter for mitochondrial stress and is a member of the DnaK/Hsp70 superfamily. The UPS assay examined a ubiquitin-related degradation signal (a “degron”) fused to CFP (P*_dat-_*_1_::CFP::CL-1). SOD-3 encodes mitochondrial superoxide dismutase. We examined oxidative stress using the transgenic line P*_sod-_*_3_::GFP as an inducible assay system. The activity of DAF-16, which is homologous to the FOXO transcription factor, was monitored using a transgenic line, P*_daf-_*_16_::DAF-16::GFP, where upregulation in response to metabolite was determined by the nuclear localization of DAF-16::GFP. For all of the assays described, upregulation is indicated with a green box, while a red box indicates no response to the metabolite using these reporter strains.

Our lab has focused on the application of the nematode, *C. elegans*, as model system whereby genetic or external factors influencing DA neuron survival can be rapidly evaluated [29]. Importantly, this model has proven to be predictive of downstream effects that have been observed in mammalian neurons, as well as genetic modifiers of neurodegeneration that have emerged in human genomic studies [30,31,32,33,34,35]. While evolutionarily distant from humans, *C. elegans* neurons retain many of the hallmarks of mammalian neuronal function. Among these, neuropeptides and neurotransmitters (dopamine, serotonin, GABA, glutamate, acetylcholine), as well as ion channel families, vesicular transporters, receptors, synaptic components, and axonal guidance molecules are highly conserved [36]. The *C. elegans* nervous system is comprised of exactly 302 neurons, eight of which produce DA (Figure 1A,B,D).

In considering the numerous advantageous attributes of *C. elegans* as a model, we employed this system to determine whether or not *Streptomyces* could cause neurodegeneration. This rapidly cultured organism (three days from egg to adult) has an experimentally accommodating lifespan (two to three weeks), and is well-suited to studies that are designed to take more exploratory concepts to mechanistic fruition rapidly and inexpensively. *C. elegans* has also been used for toxicology studies on a variety of agents that are relevant to PD, including heavy metals [37,38], pharmaceuticals [39,40,41], and ROS-inducing chemicals [42,43].

Worms eat bacteria. Indeed, *E. coli* is the standard food source that is used to maintain *C. elegans* cultures in research laboratories. Thus, we examined three common soil bacteria from the genus *Streptomyces* (*S. venezuelae*, *S. griseus*, and *S. coelicolor*). We initially attempted the direct exposure of *Streptomyces* spp. to the nematodes through feeding. Unfortunately, the worms displayed substantial aversion behavior in response to the *Streptomyces* spp. This was not too surprising, as *C. elegans* display chemosensory avoidance of unfamiliar bacteria [44]. We subsequently grew *Streptomyces* spp. (or *E. coli*, as a control) in liquid cultures to saturation and tested the resulting conditioned media for *C. elegans* DA neurodegeneration [45]. Actinomycete metabolites are typically produced during the stationary phase; therefore, *Streptomyces* spp. were sporulated and grown for two weeks in SYZ (starch, yeast extract, NZ amine) media, which is commonly used for metabolite production. Following filtration, the conditioned medium was incorporated into the nematode growth media, and animals were transferred to fresh petri dishes every two days. Strikingly, the progressive degeneration of DA neurons in worm populations was observed following exposure to *S. venezuelae* (*S. ven*) conditioned media, but not following exposure to *E. coli*, *S. griseus*, or *S. coelicolor* conditioned media (Figure 1B,C).

A notable attribute of *C. elegans* includes the ability to quantify the precise cellular complement of specific neuronal classes in genetically invariant populations. Therefore, this facilitated our capacity to evaluate sensitivity to the *S. ven* metabolite among four other neuronal subclasses, including serotonergic (5-HT), GABAergic (GABA), cholinergic (ACh), and glutamatergic (Glut) neurons [45]. Strains expressing green fluorescent protein (GFP) exclusively within these defined neuronal subclasses were scored over the course of time, specifically at four, six, and eight days of age. While DA neurons showed significant degenerative changes after four days of exposure to *S. ven* conditioned media, other neuronal classes did not exhibit degenerative changes until eight days of exposure and even at this time point, significantly more DA neurons were degenerated compared with other neuronal classes (Figure 1E). Since DA neurons exhibited enhanced vulnerability compared to other neuronal classes, we hypothesized that the presence of DA might enhance the neurodegeneration that is associated with exposure to the *S. ven* metabolite. To examine this, we exposed *cat-2* mutant worms [46] to the conditioned medium. *cat-2* worms express reduced levels of tyrosine hydroxylase (TH), the rate-limiting enzyme in the production of DA, and as a result, they contain only 40% of normal DA levels [47]. After six and eight days of exposure, *cat-2* worms displayed significantly less degeneration than wild-type worms (Figure 1E) [45]. Therefore, the presence of L-DOPA or DA might provide a sensitized cellular milieu for the *S. ven* metabolite that exacerbates neurodegeneration.

To examine if the *S. ven* metabolite causes degeneration in human cells, we used SH-SY5Y neuroblastoma cells, which is a line that is widely used in cellular models of PD. The cells were exposed to *S. ven* medium for 48 h, and cell viability was measured by the release of the intracellular enzyme, lactate dehydrogenase (LDH), and compared to the amount of LDH released by exposure to *S. coelicolor* conditioned media (which was negative for neurodegeneration in the *C. elegans* assays). Notably, dose-related toxicity was observed for *S. ven* conditioned media, but not for *S. coelicolor* [45].

We proceeded to characterize several mechanisms that are known to have a causal relationship with intracellular stress and/or neurodegeneration using *C. elegans* transgenic strains. As described in Figure 1F, we learned that the metabolite does not elicit a generalized cytoplasmic chaperone response via the hsp16/hsp20.alphaB-crystallin family of heat shock proteins [45]. Additionally, the metabolite does not upregulate the endoplasmic reticulum unfolded protein response (UPR^ER^) pathway [45]. However, it does trigger the mitochondrial unfolded protein response (UPR^MT^) pathway, and it blocks ubiquitin-related degron degradation, indicating that the ubiquitin proteasome system (UPS) is functionally impaired following metabolite exposure [43,45].

## 5. Gene-by-Environment (GxE) Interactions Modeled in *C. elegans*

**Figure 2:**
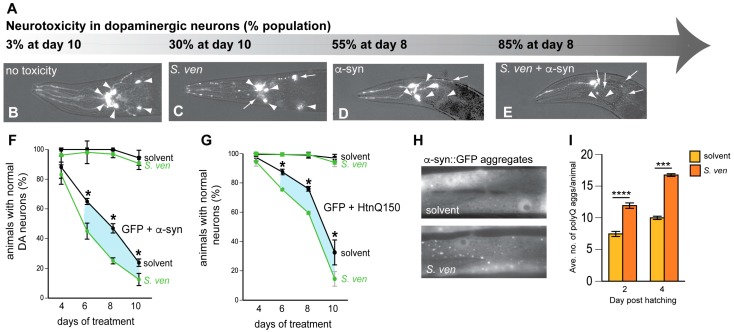
Gene-by-environment interactions exacerbate *C. elegans* phenotypes. (**A**–**E**) Genetic or environmental factors can be rapidly quantified for degenerative phenotypes by examining the six anterior dopaminergic neurons of *C. elegans*. Since the majority of cases of sporadic Parkinson’s disease (PD) are idiopathic, undefined factors from the environment and innate genetics that sensitize individuals to PD, or combinations thereof, could lower the threshold for neurodegeneration. Using *C. elegans*, the impact of both genetic and environmental exposure can be evaluated for neurotoxicity in the isogenic lines of animals for additive or synergistic effects, depending on the dosage or age of animals. (**A**) Animals displaying dopaminergic neurodegeneration can be modulated based on a causative factor; (**B**) Normal *C. elegans* rarely show dopaminergic neurodegeneration, even as animals reach old age (Day 10). Arrowheads indicate intact DA neurons; (**C**) The addition of the *S. ven* metabolite induces the age-dependent accumulation of degenerative phenotypes (arrows) and can be visualized as neuronal loss and blebbing; *S. ven* metabolite treatment alone results in ~30% neurodegeneration in contrast to animals overexpressing α-syn in the absence of *S. ven* exposure; (**D**) *S. ven* exposure in combination with a PD genetic factor, human α-synuclein (α-syn) overexpression is additive; (**E**) Whereby ~85% of the population displays neurodegeneration. Exposure to other reactive oxygen species (ROS)-inducing chemicals (i.e., 6-OHDA, rotenone, paraquat) can produce similar phenotypes; (**F**,**G**) *S. ven* exposure has been examined in combination with genetic susceptibility factors to uncover gene and environment interactions. In these scenarios, *C. elegans* with and without the expression of different heterologous aggregation-prone proteins were examined for neurotoxicity following exposure to metabolites. It should be noted that the concentration of metabolites that was used was much lower, since we wanted to ensure that, on its own, metabolite would not cause neurodegeneration, yet would reveal potential neurodegeneration in combination with α-syn [51]. The results shown here displayed this concept with either α-syn expressed in dopaminergic neurons; (**F**) or mutant huntingtin (Htn_Q150_) expressed in the *C. elegans* ASH-sensory neuron; (**G**) As shown; (**H**) The metabolite also induces α-syn-dependent proteostasis disruption in the readily visualized body wall muscle cells that express α-syn::GFP under control of a body wall muscle promoter (P*_unc-_*_54_), and were treated with *S. ven* metabolite continuously since hatching; (**I**) *C. elegans* expressing a nucleotide repeat encoding 35 polyglutatmines (Q35) in body wall muscles (P*_unc-_*_54_::polyQ35::GFP) that were exposed to the metabolite display an increase in aggregate number compared to solvent [51].

PD-associated dopaminergic neuropathology is characterized by the accumulation of α-synuclein (α-syn) in Lewy bodies. Significantly, α-syn itself can induce neurodegeneration when overexpressed or mutated [48]. The *C. elegans* genome encodes homologs of all of the familial parkinsonism genes except α-syn, which, as an autosomal dominant modulator of PD, can be overexpressed to recapitulate time-dependent degenerative pathology in vivo, including progressive DA neuron loss (Figure 2A,D) and the accumulation of misfolded protein. We were interested in determining if exposure to *S. ven* in α-syn expressing *C. elegans* would result in enhanced neurodegeneration, because there is precedent for this in the literature. For example, paraquat increases α-syn expression in mice [49]. Similarly, paraquat and rotenone also accelerate the formation of α-syn inclusions, causing a conformational change to α-syn itself, which in turn effects fibril formation [50].

Since both *S. ven* and α-syn can cause DA neurodegeneration independently, we wanted to establish chronic supplementation conditions using lower metabolite dosages in *C. elegans* with a GFP (only) marker in DA neurons so that it would no longer elicit a neurodegenerative response (Figure 2A,F). Using this sub-toxic dosing regimen, we uncovered a GxE interaction in *C. elegans* expressing α-syn in DA neurons whereby the percentage of animals with normal DA neurons was significantly decreased following six to 10 days of exposure [51] (Figure 2A–F). At day four, neurodegeneration was not enhanced, suggesting that the accumulation of α-syn is not extensive enough to manifest this neurotoxic phenotype. We performed analogous experiments with two other neuronally-expressed pathogenic proteins in vivo. One of these models expressed Aβ in the glutamatergic neurons, while the other expressed mutant huntingtin (Htn-Q_150_) in the ASH-type sensory neuron. We found that treatment with the *S. ven* metabolite similarly enhanced neurotoxicity at lower dosages (Figure 2G). These pathogenic proteins served as surrogate indictor markers of disease progression, where the dysregulation of normal homeostatic pathway function accumulates over time [52,53]. In this regard, in our three models of protein misfolding, neurodegenerative phenotypes were not observed in young animals in the absence of pathogenic protein expression, suggesting that the metabolite might synergize with threshold state animals [51]. Notably, GxE effects were not limited to misfolded proteins, as the *S. ven* metabolite similarly enhanced the toxicity of another autosomal dominant form of PD that we modeled in *C. elegans*. The LRRK2(G2019S) mutation has been shown to decrease both mitochondrial membrane potential and ATP production, resulting in neuronal toxicity [54,55]. We overexpressed LRRK2(G2019S) in DA neurons and found that *S. ven* significantly enhanced neuronal degeneration compared to solvent control (from 20% to 43% of the population with degenerating DA neurons when exposed to *S. ven* metabolite [43]). This GxE interaction was similar to a study in Drosophila where the overexpression of mutant LRRK2 (G2019S or G2385R), in combination with rotenone, caused an increase in neurodegeneration [56].

## 6. A Metabolic Fingerprint is Revealed in Response to the *S. ven* Metabolite

We wanted to assess if the metabolite-induced enhancement of neurodegeneration was correlated with alterations in protein handling. Therefore, we monitored established *C. elegans* muscle models of the overexpression of α-syn, Aβ, or polyglutamine for changes in aggregate density and/or for behavioral phenotypes following exposure to the metabolite. With all three pathogenic proteins, exposure to the *S. ven* metabolite induced phenotypic changes (Figure 2H,I, [51]).

We also have evidence that *S. ven* metabolite exposure increases reactive oxygen species (ROS) in *C. elegans* lysates using both in vivo assays and an ex vivo DCF assay from whole animal extracts [43]. An additional study examined worms expressing an oxidative stress-inducible reporter that is known to be upregulated against endogenous ROS, *sod-3*::GFP. ROS was significantly increased in these worms (Figure 1F) [43]. Since the upregulation of SOD-3 is associated with defense against oxidative stress, we treated animals expressing α-syn in either DA neurons or muscle cells with antioxidants to determine if the protein mishandling we observed was associated with oxidative damage. The four antioxidants that we tested did not attenuate phenotypes in either muscle cells or DA neurons; however, glutathione (GSH) was the only that antioxidant that suppressed both α-syn aggregate formation in muscle cells and attenuated DA neurodegeneration [51]. Through a systematic series of pharmacological and genetic studies, we determined that ubiquitin proteasome system (UPS)-linked protein homeostasis defects may result from *S. ven* exposure and that glutathione homeostasis is a regulator of metabolite-induced proteotoxicity [51].

Additional studies are required to mechanistically associate the glutathione metabolic changes with the alterations in proteostasis that we observed following *S. ven* exposure. For example, it is hypothesized that GSH attenuation occurs through the repair of damaged cysteine residues [57]. Alternatively, the glutathione couple, GSH/GSSG, might operate as a surveillance mechanism within the cellular proteome to identify redox changes within the thiol–disulfide balance of the cell [58]. Regardless of which hypothesis is correct, we suggest that shifting the GSH couple to a more reduced state might beneficially alter the neurodegenerative threshold state of *C. elegans* cells [59].

**Figure 3:**
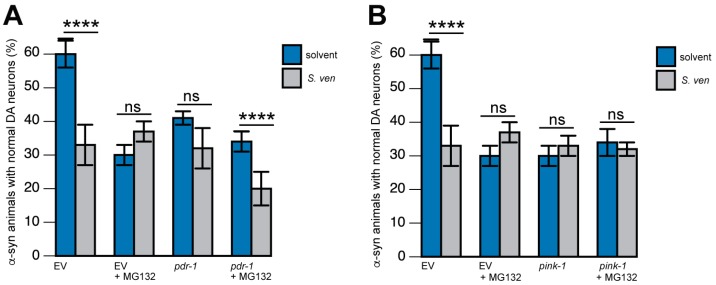
Epistatic regulation of enhanced α-synuclein toxicity by *pink-1*. (**A**,**B**) Combinations of dopaminergic stressors (such as MG132 and/or *Streptomyces venezuelae*, or *S. ven*) were applied to transgenic *C. elegans*, which was also expressed human α-syn in DA neurons. (**A**) Along with knockdown of *pdr-1* (RNAi), a more severe DA neurodegenerative phenotype was observed if compared to combinations of just two stressors at a time; (**B**) In animals where *pink-1*(RNAi) was knocked down, enhanced neurodegeneration was not observed. Instead, all of the combinations of stressors yielded similar levels of neurodegeneration with the depletion of *pink-1*, which was thereby indicative of a common mechanism revealed by putative epistatic relationships.

We also hypothesized that it was possible that the UPS perturbations and ROS induction we identified from metabolite exposure might be the result of modulating the PINK1 and/or parkin pathways. These two gene products are associated with autosomal recessive forms of PD and have been identified to regulate both protein and mitochondrial homeostasis, as well as autophagy, in many organisms [11,60,61,62,63,64]. We first depleted *pdr-1* (the *C. elegans* homolog of parkin) cell autonomously in α-syn-expressing DA neurons. Animals were treated with *S. ven* metabolite and/or the proteasome inhibitor MG132. A combination of all three stressors (α-syn, metabolite, and MG132), along with *pdr-1*(RNAi) resulted in a more severe DA neurodegenerative phenotype than any two stressors alone (Figure 3A). In contrast, when we performed a comparable experiment in α-syn animals where *pink-1* was knocked down by RNAi, increased neurodegeneration was not observed; instead, these stressors behaved in a similar manner (Figure 3B). Therefore, these data suggest that proteasome inhibition and metabolite-induced protein misfolding are epistatically regulated via *pink-1*, but are not regulated in this manner when considering *pdr-1* [51].

To further understand the mechanisms that elicit an oxidative stress response following *S. ven* metabolite treatment, we asked if the FOXO transcription factor protein, DAF-16, which is directly inhibited by the insulin signaling pathway, translocated to the nucleus following metabolite exposure. Notably, DAF-16 was translocated to the nucleus (Figure 1F) [43]; this was similar to what had been reported for paraquat exposure [65]. It is known that the nuclear accumulation of DAF-16 correlates with increased ROS. In response to this stress, the activation of genetic factors associated with pathogen defense, mitochondrial stress mechanisms, and/or cell death pathways occurs [66,67,68,69]. In this regard, we are interested in determining the targets of DAF-16 that are upregulated by metabolite exposure.

These mechanistic studies collectively reveal an emerging metabolomic fingerprint in response to the *S. ven* metabolite that share features with known toxins and the other metabolic effectors that are associated with PD, yet appear distinct in its emerging signature. As detailed below, a major component of this metabolic profile involves altered mitochondrial homeostasis.

## 7. The *S. ven* Metabolite Causes Mitochondrial Dysfunction

As previously described, following metabolite exposure the cytoprotective DAF-16 transcription factor accumulates in the nucleus and *sod-3* expression levels significantly increase. Since dysfunctional mitochondria can result in ROS and misfolded protein accumulation, we decided to explore whether a stress-response pathway referred to as the mitochondrial unfolded protein response (UPR^mt^) was triggered in response to *S. ven* metabolite exposure. The UPR^mt^ activates the transcription of mitochondrial chaperone genes to promote protein homeostasis. In *C. elegans*, *S. ven* metabolite exposure resulted in a significant upregulation of the UPR^mt^, as assayed via monitoring a nuclear-encoded mitochondrial chaperone, *hsp-6* (Figure 1F) [43]. These data are suggestive of a disturbance of mitochondrial homeostasis, especially considering that the *S. ven* metabolite does not activate the unfolded protein response signaling pathways (UPR) in the cytosol or ER (Figure 1F) [45].

The mitochondrial protein-folding environment is sensitive to alterations in organelle structure, the excess production of free radicals, and/or the improper function of the electron transport chain [70]. Therefore, while we had determined that the metabolite increased ROS, it was important to further characterize the mitochondrial phenotype associated with *S. ven* exposure. We also examined ATP levels by using an ex vivo luciferase assay with *C. elegans* extracts [43]. We determined that worms exposed to the *S. ven* metabolite displayed significantly lower overall levels of ATP compared to the solvent control; these data indicate that metabolite exposure caused the impairment of mitochondrial function. Since the structure and bioenergetics of mammalian and nematode mitochondrial respiratory chains are very similar [71], we further investigated whether the metabolite inhibited mitochondrial complex I in a manner similar to rotenone, an environmental toxin that also causes DA neurodegeneration in *C. elegans* [10,72]. Furthermore, we elected to evaluate the activity of two specific compounds for their capacity to protect against the DA neurodegeneration that was caused by *S. ven* exposure. In this regard, we examined the treatment of *C. elegans* with riboflavin, which is a mitochondrial complex I (NADH dehydrogenase) activator, and D-beta-hydroxybutyrate (DβHB), which is an activator of mitochondrial complex II (succinate dehydrogenase) that can rescue complex I deficiencies via a mechanism dependent on complex II function [73,74]. Both riboflavin and DβHB treatment significantly rescued *S. ven* neurotoxicity [43].

We also wanted to determine if the metabolite altered mitochondrial membrane potential (ΔΨ_m_); therefore, we exposed worms to *S. ven*, and then measured the relative mitochondrial uptake of the fluorescent dye tetramethylrhodamine ethyl ester (TMRE), which accumulates in active mitochondria. Live animals exposed to metabolite displayed significant decreases in TMRE fluorescence, thus demonstrating that the metabolite is associated with ΔΨ_m_ collapse.

## 8. The *S. ven* Metabolite Disrupts Mitochondrial Homeostasis

**Figure 4:**
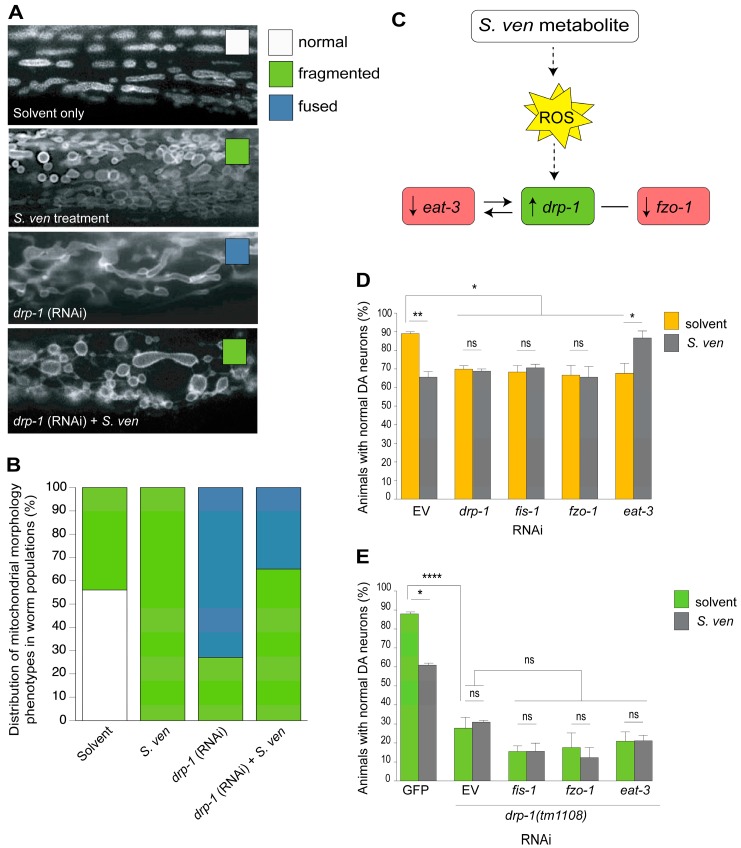
*S. ven* metabolite increases mitochondrial fragmentation. The impact of the *S. ven* metabolite on the mitochondrial outer membrane in *C. elegans* body wall muscle cells in animals expressing an outer mitochondrial protein targeted RFP (P*_myo_*_3_::TOM20::mRFP). (**A**) Compared with solvent treatment, *S. ven* metabolite-treated animals exhibit significantly more mitochondrial fragmentation following exposure (green box). RNAi knockdown of *drp-1*, an outer mitochondrial membrane fission gene, reveals significantly increased fusion in these animals (blue box). Notably, in *drp-1* (RNAi) worms treated with *S. ven*, many animals return to a fragmented mitochondrial phenotype. Mitochondrial morphology is defined as normal (tubular—white box), fused (elongated—blue), or fragmented (circular and irregular—green); (**B**) Quantitation of mitochondrial morphology phenotypes in *C. elegans* populations. The distribution of fragmented mitochondria is different between all of the samples. In *S. ven* and *drp-1* RNAi + *S. ven* treated populations, increased fragmentation is indicative of damaged mitochondria that cannot be turned over by mitophagy [83]. The color scheme (white, blue, green) is the same as shown in (**A**); (**C**) Schematic representation of qPCR data showing that *S. ven* metabolite exposure in *C. elegans* leads to increased *drp-1* gene expression, as well as lowered *fzo-1* and *eat-3* gene expression [83]. DRP-1 is an outer mitochondrial membrane fission protein, while FZO-1 and EAT-3 are fusion proteins that are located at the outer and inner mitochondrial membranes, respectively; (**D**) *eat-3*(RNAi) knockdown suppresses dopaminergic neurotoxicity caused by the metabolite. Notice that the knockdown of all of the other mitochondrial fission and fusion genes still causes toxicity in the presence of metabolite, except for *eat-3* (RNAi), which now exhibits neuroprotection; (**E**) The RNAi sensitive strain used in part D (above) was crossed to *drp-1* loss-of-function mutant (allele *tm1108*) animals. In this background, all of the fission and fusion genes that were examined display enhanced sensitivity to neurodegeneration, notably, *eat-3* (RNAi*); drp-1(tm1108)* no longer shows resistance in the presence of the metabolite.

Since abnormal mitochondrial fission/fusion is associated with ΔΨ_m_ collapse [75,76], we decided to explore whether the metabolite altered the regular fission/fusion cycles that are regulated by the GTPases (Drp1 and Opa1) and mitofusins (Mfn1/Mfn2) that are located on the outer and inner mitochondrial membranes. Furthermore, it is well-established that other environmental toxins alter mitochondrial homeostasis [77,78,79,80,81]. We determined that the *S. ven* metabolite increases mitochondrial fragmentation/fission, as visualized in the large body wall muscle cells of *C. elegans*, in a time-dependent manner, whereby animals are more sensitive to the metabolite as they age (Figure 4A,B). Additionally, there is a concomitant decrease in *fzo-1* gene expression and an increase in *drp-1* gene expression (outer mitochondrial fusion and fission genes, respectively) (Figure 4C). Comparable with results from studies that have been conducted using human cells in culture, our worm data revealed that mitochondrial fragmentation resulted from mitochondrial oxidative stress due to an imbalance in Mfn2 and Drp1 activity [82]. In *C. elegans*, *drp-1* and *fzo-1,* the outer mitochondrial membrane genes have changes in expression following treatment with the *S. ven* metabolite.

We proceeded to compare the gene expression profiles obtained from *S. ven* exposure to those obtained from other environmental PD toxins. While *S. ven* enhances mitochondrial fragmentation, rotenone decreases fission and induces fusion with an associated decrease in *Drp1* gene expression [77,78,81]. Conversely, MPP^+^ increases mitochondrial fragmentation and increases Drp1 protein levels, which is similar to *S. ven* [79,80]. However, when human *Drp1* is genetically inactivated in SH-SY5Y cells, this blocks MPP^+^ mitochondrial fragmentation [79]. Whereas in an analogous experiment in *C. elegans*, when *drp-1* is knocked down, fragmentation still occurs following treatment with *S. ven* [83]. Thus, the mitotoxic mechanisms of action are clearly distinguishable among these substances, even though all three inhibit mitochondrial complex I.

Since metabolite exposure resulted in pronounced mitochondrial morphological changes, we also examined how modulating fission/fusion impacted DA neurodegeneration. We utilized a RNAi strain that allows for selective RNAi knockdown exclusively in DA neurons to examine mitochondrial fission/fusion components following the exposure of the metabolite [84]. There was significant DA neurodegeneration following RNAi depletion for fission (*drp-1* and *fis-1*) and fusion (*fzo-1* and *eat-3*) genes when compared to solvent-only empty vector (EV) control (Figure 4D). Following the addition of metabolite, neurodegeneration was enhanced in EV, but no further degeneration was observed in *drp-1*, *fis-1,* or *fzo-1* RNAi knockdown (Figure 4D) [83]. In contrast, the OPA1 homolog, *eat-3*(RNAi) and metabolite revealed a resistance to DA neurotoxicity (Figure 4D). The *eat-3* data seemed curious until we found a previous publication reporting that *drp-1*, an outer mitochondrial fission component, can act genetically upstream of *eat-3*, which is an inner mitochondrial fusion component [85]. The report further suggested that there is mutual compensation for physiological defects. In this regard, we considered our own data where *S. ven* metabolite increases *drp-1* gene expression levels (Figure 4C) and hypothesized that we could reverse the resistance to DA neurodegeneration that occurs in an *eat-3* background by depleting both *eat-3* and *drp-1* in the same animals. Therefore, using our DA neuron-selective RNAi strain to test the putative interaction between *eat-3* and metabolite-induced *drp-1*, the effect of *eat-3* (RNAi) on DA neurodegeneration in a *drp-1 (tm1108)* null mutant background was evaluated. We observed no neuroprotection against the *S. ven* metabolite in the *drp-1* null mutant background. Thus, we surmised that there is an epistatic regulatory relationship between the metabolite-induced *drp-1* activity depletion of *eat-3* [83] Figure 4E). When *fis-1* or *fzo-1* were knocked down in the *drp-1* mutant background, further neurodegeneration did not occur (Figure 4E). The interdependence between *drp-1* and *eat-3* was further confirmed with qPCR (data not shown; Figure 4C; [83]).

## 9. PINK-1/DRP-1-Dependent Fission Induced by *S. ven* Metabolite

PINK1 and parkin often function together to promote DRP-1-dependent mitochondrial fission [86,87]. In exploring this functional relationship in the context of the *S. ven* metabolite, we postulated that if metabolite-induced fission is independent of PINK-1 activity, it would suppress DA neurodegeneration in *drp-1* mutants. Conversely, when *pink-1* was depleted, we predicted that treatment with the *S. ven* metabolite would not enhance neurodegeneration if *drp-1* was dependent on *pink-1* function. Indeed, we discerned that the DA neurodegeneration that was caused by metabolite exposure was not further enhanced by *dpr-1* RNAi in the absence of *pink-1*. Therefore, DRP-1 activity in mitochondrial fission appears to be required for metabolite-induced DA neurodegeneration in a *pink-1* mutant background. We also discerned that the reduction of *eat-3* (RNAi) significantly suppressed *pink-1*-induced neurodegeneration with or without metabolite exposure. Similar results were obtained when examining a *pdr-1* loss-of-function mutant. These data indicate that downregulation of *eat-3* is neuroprotective in *pink-1* or *pdr-1* mutant conditions [83].

Following mitochondrial dysfunction, an increase in DRP-1 activity can occur through AMP-activated protein kinase (AMPK), which is a key regulator of energy metabolism [88,89]. AMPK can be activated by rotenone and antimycin A and can promote mitochondrial division [88]. In this regard, we wanted to determine if the *S. ven* metabolite was also associated with this type of response. We performed both genetic and pharmacological studies, and assayed DA neurodegeneration in *C. elegans*. AMPK suppressed metabolite-induced DA neurotoxicity in N2 wildtype, *eat-3* (RNAi), and *pink-1*; *drp-1*(RNAi) animals. From these results we concluded that AMPK plays a mechanistic role in *S. ven* metabolite-induced DA neurodegeneration, although more research will be required to determine how AMPK activity is modulated [83].

## 10. Toward the Identification of the Neurotoxic Metabolite

In most of our previously described assays, the *S. ven* metabolite was partitioned sequentially through DCM, water, and chloroform to provide an enriched form, which we know from thin-layer chromatography and bioassay testing contains six fractions, two of which cause neurodegeneration. This neutral-lipid fraction is dried down, resuspended in ethyl acetate, and tested for DA neuronal death in *C. elegans* assays. When the activity is confirmed, a concentration that is appropriate for neuronal death equivalents/mL of metabolite is established. The metabolite is then incorporated into Petri dishes for use in culturing/exposure to *C. elegans*.

Our goal is to identify the chemical structure of the neurotoxic molecule and then chemically synthesize it for long-term experimental use. Fractionation-guided purification was performed, starting from spent *S. ven* media, whereby it was subjected to reverse-phase semi-preparative HPLC. The resulting fractions were analyzed using the *C. elegans* DA neurodegeneration assay, and one fraction was found to be significantly active. High-resolution electrospray ionization mass spectrometry (HRESIMS) and nuclear magnetic resonance (NMR) analysis permitted elucidation of the structure, which is novel. Current efforts are underway for compound identification via proof-of-structure by chemical synthesis. The authors are not comfortable prematurely revealing structural information until this “gold standard” for biomolecule identity has been achieved.

## 11. Exposure to Other Soil *Streptomyces* Species also Causes Neurodegeneration

**Figure 5:**
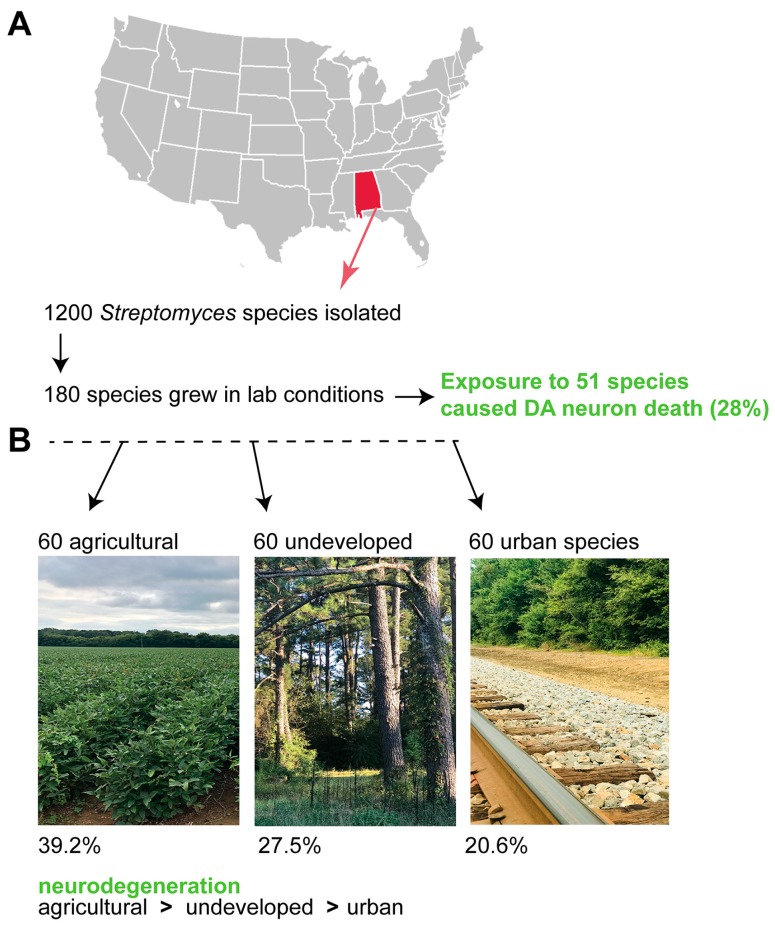
*Streptomyces* isolates from the environment cause DA neuron death in *C. elegans*. (**A**) We isolated 1200 *Streptomyces* from diverse regions of Alabama, of which 180 samples were grown in laboratory conditions [90]. Of these, 51/180 resulted in significant DA neuron death (28%); (**B**) These same 180 samples that grew in the lab represented isolates from three diverse locations: agricultural soils, urban soils, and pristine/undeveloped areas. We examined whether rates of neurodegeneration varied according to sample location, and learned that there were significant differences, with all three soil types exhibiting significant differences in neurodegeneration from each other. Agricultural soils caused the most degeneration, and urban soils caused the least amount of neurodegeneration [90]. Exemplar images of the areas that were sampled are displayed here.

All of the research described within this review was performed with *Streptomyces venezuelae*, which is an American Type Culture Collection isolate. However, given the ubiquitous distribution of *Streptomyces* species (spp.) within the soil, either chronic or acute exposure to this metabolite could represent a previously unreported contributor to the onset of neurodegeneration, and may be exacerbated by factors influencing genetic pre-susceptibility to neurodegeneration. Therefore, we proceeded to conduct a regional microbial ecology survey of 1200 natural *Streptomyces* spp. from diverse land uses in the state of Alabama; 180 of these soil samples grew in laboratory conditions (Figure 5). From these, we learned that 51 of the species (28%) caused significant DA neuron death in *C. elegans* [90].

The species that grew in lab conditions could be further subdivided into land-use patterns (agricultural, undeveloped, or urban soils; Figure 5). Notably, there was significant differences in neurodegeneration among all three soil types, with 39.2% of agricultural *Streptomyces* spp., 27.5% of undeveloped *Streptomyces* spp., and only 20.6% of urban spp. causing neurodegeneration [90]. These data suggest that there could be a common environmental toxicant(s) within the *Streptomyces* genus that causes neurotoxicity. In this regard, it is common for multiple species within a bacterial genus to produce related metabolites [91,92]. Understanding how distinctions in microbial ecology might intersect with the socio-economic disparities that impact health with respect to neurodegenerative diseases of aging represents a major unmet medical challenge of our time.

## 12. Summary and Future Studies

We have described the phenotypic consequences in *C. elegans* that are associated with exposure to bacterial lipophilic and amphipathic secondary metabolites by chemically extracting post-log bacteria cultures of the soil bacterium *S. venezuelae*. In a broad sense, *S. ven* exposure in *C. elegans* mirrors some of the pathological hallmarks of idiopathic PD, including ubiquitin proteasome system (UPS) disruption, glutathione homeostasis perturbation, general perturbation of proteostasis, mitochondrial dysfunction, and mitophagic alteration [93,94,95]. These observations are suggestive of the following two points about *S. ven* exposure.

First, *S. ven* metabolite toxicity mimics idiopathic PD in a way that is reminiscent to other environmental compounds that also cause stress in broad, diverse, cellular pathways. The cellular response to *S. ven* exemplifies the concept whereby genetic pathways function as interactome networks [96,97]. In these networks, distant genetic components (UPS, mitochondrial dysfunction, etc.) are eventually directed into large, central, organizing pathways (for example, mitophagy). Thus, peripheral pathway dysfunction will eventually lead to central pathway dysfunction, and widespread cellular failure will occur. As such, the pathology of idiopathic PD may arise from the collapse of interconnected pathways with time.

In the future, we plan to perform a metabolomic profile analysis in *C. elegans* to identify the small molecules that influence the cellular dysfunction associated with *S. ven* exposure. Our interest in profiling the metabolome is a direct result of data showing that glutathione directly modulates α-syn-induced neurodegeneration and misfolding. Metabolomic profiling has successfully identified metabolic differences in *C. elegans* through studies examining natural variation in populations, as well as in an analysis of longevity and another of transgenic amyloid-beta expression [98,99,100]. We also intend to profile the transcriptome following exposure to the *S. ven* metabolite. As described in Section 6, *C. elegans* DAF-16 (the FOXO transcription factor) is translocated to the nucleus following metabolite exposure [43]. Based on our prior results, we predict that the transcriptome will include gene products that are modulated by DAF-16 and/or the UPR^mt^ in addition to illuminating previously unattained regulators. After we obtain data from both the metabolomic and transcriptomic platforms, we plan to superimpose these data in a cross-omics approach, as is often performed to identify network interactions [98]. This dual analysis will allow us to capture changes occurring at two regulatory levels and pinpoint common pathways of cellular dysfunction that occur following *S. ven* exposure. We also have an interest in further exploring metabolite-induced cellular dysfunction as it pertains to mitochondrial biogenesis. Here, we will explore AMPK activity as a means to modulate *drp-1* and *eat-3* gene transcription levels and examine their impact on mitochondrial activity and dysfunction.

Second, the environment is replete with damaging toxins from either bacteria or other natural sources. For example, as we have shown from a recent collaborative study in our lab, many other *Streptomyces* spp. secondary metabolite products display neurodegenerative potential [90]. Prokaryotic organisms represent an overwhelming majority of life on earth [101]. Despite the enormous diversity of bacteria, their relationship with Eukarya is not yet well described. Therefore, their native environments, and biochemical and cellular interactions, may well provide us with a wealth of future information. Thus, it is intriguing to consider the secondary metabolites that have been excreted from these soil bacteria as a potentially large source of environmental stress. Future studies could include examining various environmental conditions, such as rotenone or paraquat exposure, on soil *Streptomyces* spp. for their impact on toxin production.

In summary, the *Streptomyces venezuelae* metabolite causes cellular stress in a manner that is similar yet distinct from other PD environmental toxins. It is this unique signature, and prevalence of this genus within the environment, that makes this metabolite an intriguing molecule for further investigation. It is tempting to envision chronic exposure to such a factor as an unforeseen environmental component impacting long-term neuronal survival during the human aging process. Given our increasingly aging global population, the potential ramifications of identifying a causal, or even contributory, environmental factor for neurodegeneration is substantial. This information would open a door toward the identification of factors controlling disease susceptibility, which could be used to gauge or reduce environmental risk and serve to accelerate the development of novel treatment strategies.
